# Mutational Biases and GC-Biased Gene Conversion Affect GC Content in the Plastomes of *Dendrobium* Genus

**DOI:** 10.3390/ijms18112307

**Published:** 2017-11-02

**Authors:** Zhitao Niu, Qingyun Xue, Hui Wang, Xuezhu Xie, Shuying Zhu, Wei Liu, Xiaoyu Ding

**Affiliations:** College of Life Sciences, Nanjing Normal University, Nanjing 210023, China; niuzhitaonj@163.com (Z.N.); qyxue1981@126.com (Q.X.); wanghui201711@163.com (H.W.); naive0312@126.com (X.X.); zhushuy@126.com (S.Z.); liuwei4@njnu.edu.cn (W.L.)

**Keywords:** *Dendrobium*, plastome assembly, selection, mutational biases, GC-biased gene conversion (gBGC), GC_eq_

## Abstract

The variation of GC content is a key genome feature because it is associated with fundamental elements of genome organization. However, the reason for this variation is still an open question. Different kinds of hypotheses have been proposed to explain the variation of GC content during genome evolution. However, these hypotheses have not been explicitly investigated in whole plastome sequences. *Dendrobium* is one of the largest genera in the orchid species. Evolutionary studies of the plastomic organization and base composition are limited in this genus. In this study, we obtained the high-quality plastome sequences of *D. loddigesii* and *D. devonianum*. The comparison results showed a nearly identical organization in *Dendrobium* plastomes, indicating that the plastomic organization is highly conserved in *Dendrobium* genus. Furthermore, the impact of three evolutionary forces—selection, mutational biases, and GC-biased gene conversion (gBGC)—on the variation of GC content in *Dendrobium* plastomes was evaluated. Our results revealed: (1) consistent GC content evolution trends and mutational biases in single-copy (SC) and inverted repeats (IRs) regions; and (2) that gBGC has influenced the plastome-wide GC content evolution. These results suggest that both mutational biases and gBGC affect GC content in the plastomes of *Dendrobium* genus.

## 1. Introduction

Chloroplasts, responsible for photosynthesis and other biosynthesis processes in plants, have essential effects on plant growth and development. Their own genomes (plastomes) are usually uniparentally inherited and highly conserved in their quadripartite structure, which consists of a pair of inverted repeats (IRs) regions and two single-copy (SC) regions [1]. Comparative studies of the plastome sequence have revealed: (1) a relatively higher GC content in IR regions than that of SC regions and (2) varied GC content in different gene and non-coding loci e.g., [2,3]. The variation of GC content is a key genome feature because it is associated with fundamental elements of genome organization [4,5]. For instance, GC-rich regions exhibit higher gene density, more conserved mutation rates, and higher recombination rates, relative to GC-poor regions. Therefore, resolving the origin and causes of the variation in base composition has practical significance for a better understanding of the plastome organization.

Three major kinds of hypotheses have been proposed to explain the variation of GC content in genome evolution. The first hypothesis, “natural selection hypothesis”, has suggested that high GC content can be selected for by their thermal stability [4,6]. Natural selection also affects the probability of fixation of a mutation based on the mutation fitness advantage or disadvantage of the organism [7,8]. The second hypothesis, the so-called “mutational biases hypothesis”, is that the GC content is driven by the heterogeneous mutational biases along genomes [9]. The third hypothesis involves GC-biased gene conversion (gBGC), a process that takes place during meiotic recombination. The gBGC process prefers repairing DNA mismatches with GC bases and tends to increase the GC content of recombining DNA over evolutionary time [10,11]. This was recently confirmed by a broad range of genome comparison studies in eukaryotes and prokaryotes [12,13,14]. For example, in yeast genomes, gBGC occurred to repair the mismatches located at the extremities of the conversion regions [15]. The gBGC was also demonstrated to affect the GC content of third codon position and intron regions in grasses genomes [16]. Recently, Wu and Chaw, 2015 measured the gene conversion events of non-coding regions in cycads plastomes and reported the first case of plastome GC-biased gene conversion [17].

However, these hypotheses have not been explicitly investigated in whole plastome sequences. Orchids (Orchidaceae) are the largest family in the monocots, including about 25,000 species in 880 genera and five subfamilies [18]. Recent studies showed a diversified evolution of the plastome sequence among different orchid genera [19,20]. Moreover, orchids also present a peculiar plastomic structure: they exhibit variable IRs that caused the drastic reductions in small single-copy (SSC) regions (e.g., the expansion/contraction of IRs has led to the length of the SSC region in *Vanilla* being only about one-eighth of that in *Goodyera*) [20]. These features result in a variable GC content among different plastome sequences. However, there is still little known about the GC content evolution of the orchid plastome sequences.

Recently, plastome sequences have been made available for more than 20 orchid genera. However, in this study, we chose to focus on the *Dendrobium* genus because it is the only genus of orchids for which more than 30 plastome sequences have been sequenced [21,22]. *Dendrobium* is one of the largest genera in the orchid species. In China, there are about 80 *Dendrobium* species, some of which are well known for their high horticultural and medicinal value [23,24]. Although many plastome sequences have been published, the research of plastomic structure and base composition remains very limited in the *Dendrobium* species. In this study, we surveyed 10 plastomes of *Dendrobium* species, including two newly sequenced ones, and we addressed two key questions: (1) Is the plastome structure conserved among *Dendrobium* species or variable due to the expansion/contraction of IRs? (2) Which evolutionary forces—selection, mutational biases, or gBGC—have a significant impact on GC content in orchid plastomes?

To address the questions mentioned above, the complete plastome of two more *Dendrobium* species (*D. loddigesii* and *D. devonianum*) were sequenced and assembled by two different methods. The plastomic structures among *Dendrobium* plastomes were compared. Moreover, we evaluated the selection forces among the plastid protein-coding genes of 10 *Dendrobium* species. Meanwhile, biased mutations of protein-coding genes and non-coding regions were also measured on the basis of the estimated equilibrium GC content (GC_eq_). Our results suggest that both mutational biases and gBGC affect GC content in the plastomes of the *Dendrobium* genus.

## 2. Results

### 2.1. Plastome Assembly of D. loddigesii and D. devonianum

A total of approximately 3.84 Gb of 150 bp pair-end reads each for *D. loddigesii* and *D. devonianum* was obtained from the Illumina paired-end sequencing. Reads with error probability >0.05 were discarded. After that, the plastomes of *D. loddigesii* and *D. devonianum* were assembled by two different ways: (1) de novo assembly by using SOAPdenovo version 1.12 and (2) using the reference-guided mapping method with CLC Genomics Workbench 6.0.1. For the de novo assembly analysis, 43,509 contigs of *D. loddigesii* and 32,667 contigs of *D. devonianum* were included in the initial assembly. After comparison with plant plastomes, 88 contigs and 67 contigs were obtained with E-values <10^−10^ and average coverage depth >30× for *D. loddigesii* and *D. devonianum*, respectively. Six of these contigs (length >17 kb and coverage depth >117×) resulted in a nearly complete draft genome for *D. loddigesii*. Four contigs (length >25 kb and coverage depth >172×) were employed for the plastome assembly of *D. devonianum*. After assembly and gap closure, the plastome sequences of *D. loddigesii* and *D. devonianum* were 152,874 bp and 152,215 bp in length, respectively. For the reference-guided mapping analysis, the trimmed reads were mapped to the plastome sequence of *D. moniliforme* (AB893950), which served as a reference sequence. After gap closure, we obtained the plastome sequences of *D. loddigesii* and *D. devonianum* with 149,674 bp and 150,973 bp in length, respectively.

The assembled plastomes of *D. loddigesii* and *D. devonianum* were compared and plotted using mVISTA, with *D. moniliforme* as the reference (Figure S1). The comparison results showed that the differences—including single nucleotide polymorphism (SNP), insertion-deletion (InDel), and sequence repeat—between the plastomes were mainly distributed in the non-coding regions and *ndh* pseudo genes. Each type of these errors was corrected and validated by PCR amplification and Sanger sequencing. After that, the complete plastomes of *D. loddigesii* and *D. devonianum* were obtained with 152,498 bp and 151,715 bp respectively.

### 2.2. Highly Conserved Plastomic Structure and Organization in Dendrobium Genus

**Figure 1:**
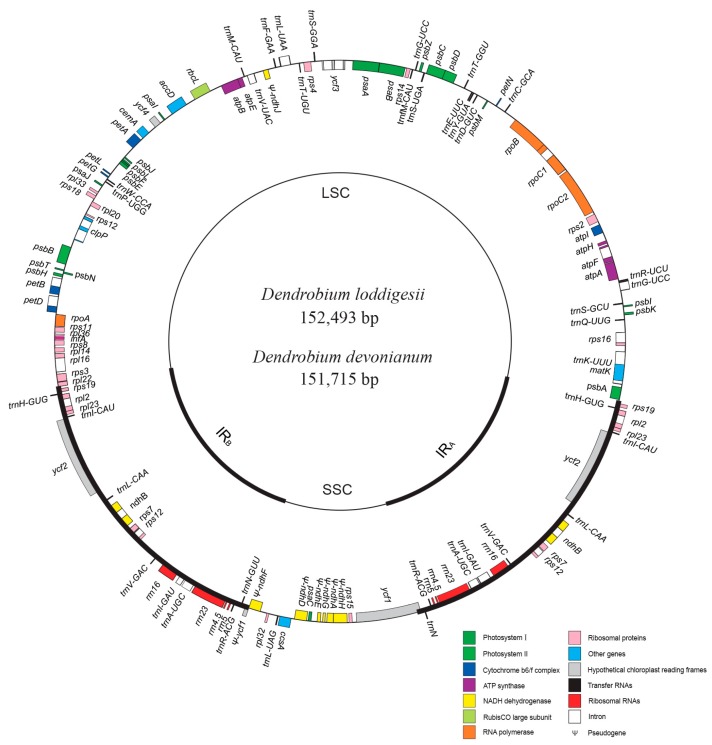
Genome map of two newly sequenced *Dendrobium* plastomes. Only the plastome of *D. loddigesii* is shown because it has an identical structure with *D. devonianum*. Genes outside and inside the circle are transcribed clockwise and counterclockwise, respectively. LSC: large single-copy; SSC: small single-copy; IR_A_ and IR_B_: two identical inverted repeats.

Genome maps of the newly sequenced two *Dendrobium* plastomes, including *D. loddigesii* and *D. devonianum*, were circular, as shown in Figure 1. The plastome contained a pair of inverted repeat (IR) regions, which separated the single-copy (SC) region into large SC (LSC) and small SC (SSC) regions. The LSC, SSC, and IR regions of *D. loddigesii* and *D. devonianum* ranged from 84,089 to 84,897 bp, 14,311 to 16,932 bp, and 25,736 to 25,800 bp, respectively. The overall GC content (37.38–37.56%) was similar with other *Dendrobium* plastomes [20,22], with 35.12–35.36% and 30.54–30.61% in LSC and SSC regions, respectively, and a higher content of 43.51–43.57% in the IR regions (Table 1). The two *Dendrobium* plastomes contained 102 unique functional coding genes, including 30 tRNA genes, four rRNA genes, and 68 protein-coding genes.

**Figure 2:**
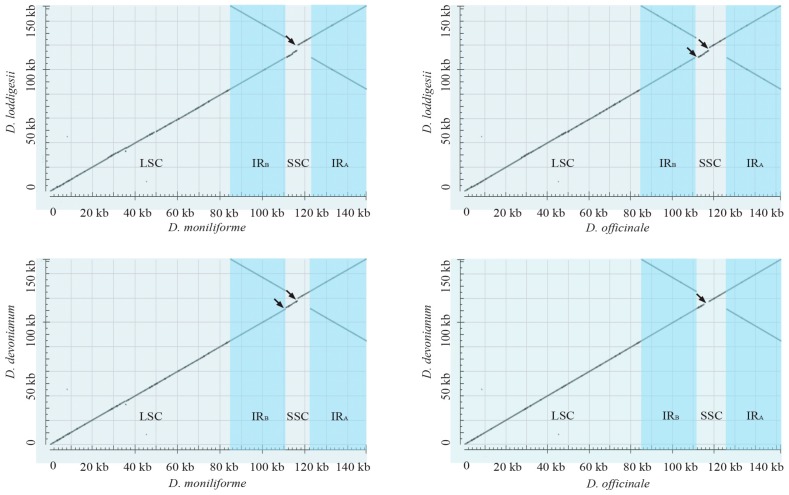
Dot-plot analysis of the four *Dendrobium* plastomes. The *Dendrobium* plastomes appear to have a nearly identical organization, which indicates that their plastomic organization is highly conserved. The black arrows indicate the different loss/retention of *ndh* genes.

The plastome sequences of *D. loddigesii*, *D. devonianum*, *D. officinale*, and *D. moniliforme* were used for the plastome comparison (Figure 2). Comparative plastomes of these four *Dendrobium* species revealed distinct loss or retention of *ndh* genes, which indicated that the *ndh* genes have experienced independent loss during their evolution. Compared to the variable expansion/contraction of IRs in different orchid genera, e.g., [20,21], the IRs of plastomes in the *Dendrobium* genus were conserved. Overall, these *Dendrobium* plastomes appeared to have a nearly identical organization reflecting the highly conserved plastomic organization in the *Dendrobium* genus.

### 2.3. Mutational Biases of Non-Coding Loci Are Associated with the Plastomic Structure 

**Figure 3:**
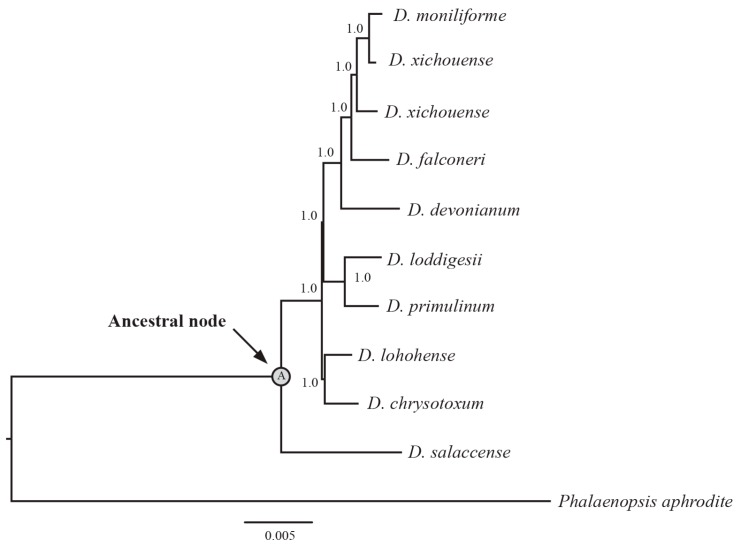
The BI tree inferred from whole plastome sequences of *Dendrobium* species. Tree node labeled with “A” denotes to the ancestor for each *Dendrobium* species. The values of posterior probabilities are showed for each node.

The phylogenetic relationship of these 10 *Dendrobium* species was inferred from the whole plastome sequence (Figure 3). The phylogenetic tree was highly resolved with the support value of all nodes = 100%. This tree was utilized to construct the ancestral sequences of non-coding loci, including intergenic and intronic loci for the 10 *Dendrobium* species. Then, the point mutations between ancestral and current sequences were calculated.

**Figure 4:**
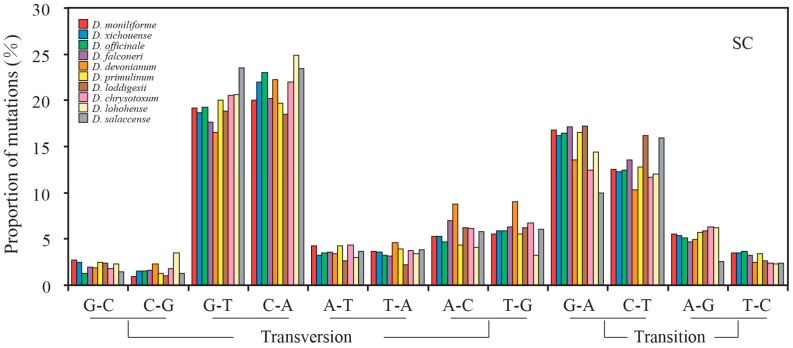

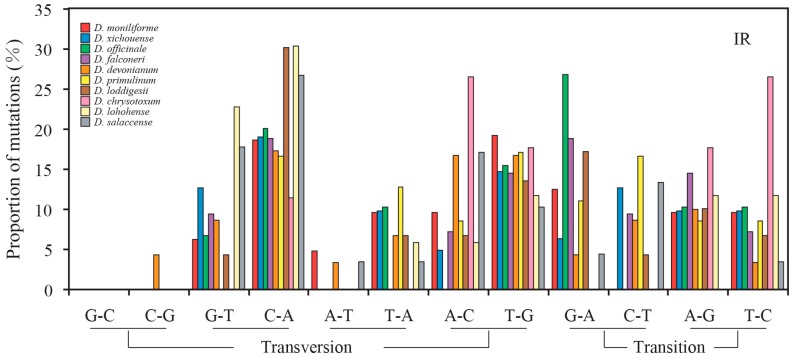
Proportion of the six nucleotide-pair mutations estimated from non-coding loci of the SC and IR regions of 10 *Dendrobium* species. The numbers of A to G mutations are normalized for the unequal nucleotide content of *Dendrobium* species. The frequencies of transversions are higher than that of transitions in both SC and IR regions.

**Figure 5:**
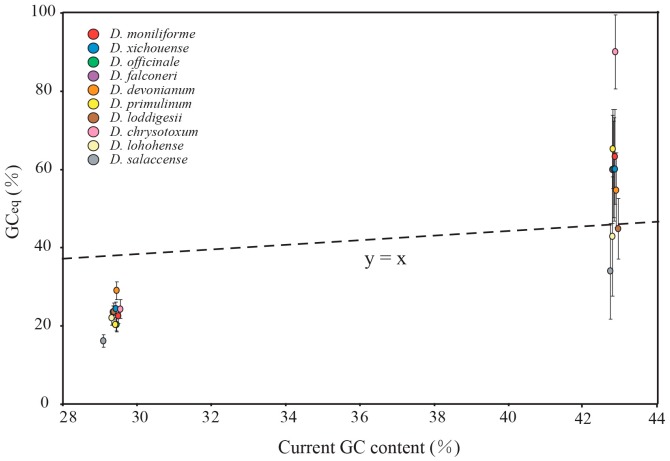
Comparison of equilibrium GC content (GC_eq_) values between the SC and IR regions for the non-coding regions. Error bars depict 95% confidence intervals for GC_eq_. Note that, the non-coding regions showed contrast GC_eq_ values (<50% in SC and >50% in IR) in SC and IR regions. Moreover, the estimated GC_eq_ values are lower than current GC content in SC regions, but higher than current GC content in IR regions.

In all examined *Dendrobium* species, the nucleotide mutations were mainly distributed in the non-coding loci of SC regions. The frequencies of transversions are higher than that of transitions in both SC and IR regions. Figure 4 shows the relative rates of the six nucleotide pair mutations. The most common mutation is G/C to A/T mutations in SC regions. Therefore, we divided the nucleotide mutations into two groups: AT-rich (G/C to A/T) and GC-rich (A/T to G/C) mutations (Table 2). The number of AT-rich mutations was larger than that of GC-rich mutations in the SC regions, while the two types of mutations did not display significant difference in the IR regions. These results indicated that the mutational biases in *Dendrobium* plastomes are associated with the plastomic structure. Moreover, the contrasting biased mutations between the SC and IR regions were also evident by the GC_eq_ values estimated from the non-coding loci of the SC and IR regions. In line with the counting results shown in Table 2, the GC_eq_ values for the SC regions were remarkably smaller than equilibrium (GC_eq_ = 50%) (Figure 5). However, in the IR regions, GC_eq_ values of all *Dendrobium* species were greater than 50%, except for *D. loddigesii*, *D. lohohense*, and *D. salaccense* (with GC_eq_ values 44.83%, 42.86% and 33.97%, respectively). This result disagreed with the data shown in Table 2, that the IR regions for *Dendrobium* species have no significant difference between GC-rich and AT-rich mutations. Because only few mutations were detected in the IR regions, the data shown in Table 1 might not precisely reflect the mutational biases of non-coding loci. Thus, based on the estimated GC_eq_ values, the mutational biases in the IR region were toward GC-richness. Considering the different mutational trends in *Dendrobium* plastomes: (1) toward AT-richness in SC regions and (2) toward GC-richness in IR regions, we proposed that the mutational biases of non-coding loci are associated with the plastomic structure in *Dendrobium* species.

### 2.4. Contrasting Mutational Biases of Protein-Coding Genes between SC and IR Regions

**Figure 6:**
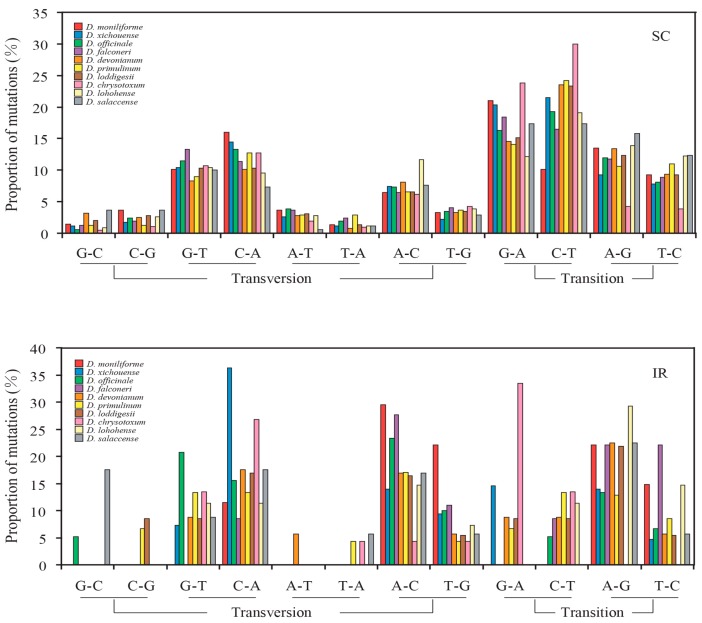
Proportion of the six nucleotide-pair mutations estimated from protein-coding genes. In contrast to the counting results of non-coding loci, the frequencies of transitions are higher than that of transversions in both SC and IR regions.

**Figure 7:**
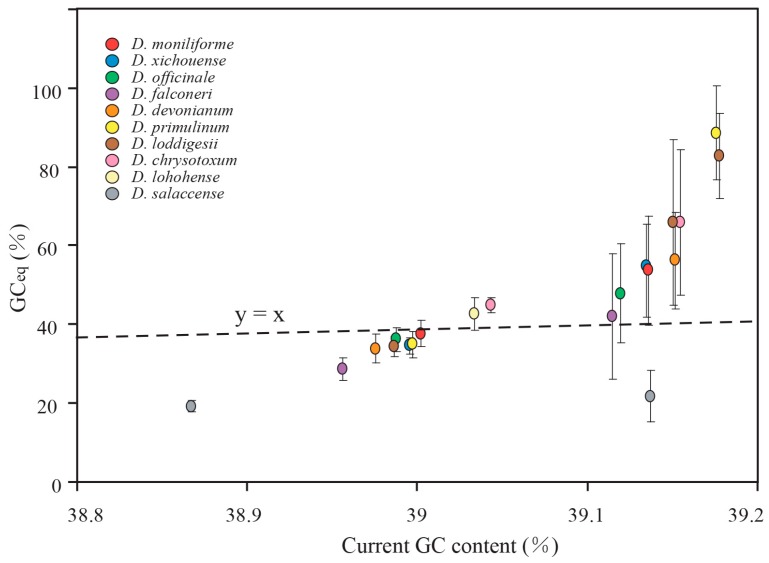
Comparison of GC_eq_ values between the SC and IR regions for the protein-coding genes. Error bars depict 95% confidence intervals for GC_eq_. The protein-coding genes showed the same evolution trends with the non-coding loci.

Three pairs of site models (M0 vs. M3, M1a vs. M2a, and M7 vs. M8) were used to test whether the evolution of plastid protein-coding genes was driven by positive selection. Among 68 plastid protein-coding genes, twelve genes (*accD*, *ccsA*, *matK*, *psaB*, *rbcL*, *rpl20*, *rpoC1*, *rpoC2*, *rps3*, *rps16*, *ycf1* and *ycf2*) were detected under positive selection (Table S1). The plastid protein-coding genes were classified into two categories, positive selected and non-positive selected data sets. The mutational biases were counted. However, the two data sets showed the same mutational trends: (1) the frequencies of transitions were higher than that of transversions (Figure S2); (2) counts of AT-rich mutations were larger than that of GC-rich mutations (Table S2); and (3) the GC_eq_ values were smaller than equilibrium (GC_eq_ = 50%) (Figure S3). Similar results were also observed in each codon position, which suggests that positive selection has no effect on gene conversions. Therefore, the plastid protein-coding genes were re-divided into SC and IR data sets based on their locations. The genes of *rpl22*, *rps12*, and *ycf1* were discarded because they were distributed in both SC and IR regions. Consistent with the counting results of non-coding loci, protein-coding genes also showed a contrast mutational bias between SC and IR regions (Table 3 and Figure 6 and Figure 7).

### 2.5. Mutational Biases Have Directly Impact on the Evolution of GC Content in Dendrobium Plastomes

As mentioned above, our analysis revealed contrast mutational biases between SC and IR regions in both protein-coding genes and non-coding loci. To assess the effect of mutational biases on the evolution of GC content in *Dendrobium* plastomes, the GC_eq_ values were compared to current GC content. As shown in Figure 5 and Figure 7, the GC_eq_ values of protein-coding genes and non-coding loci in SC regions were estimated to be 19.25–44.88% and 16.05–28.92%, respectively. The estimated GC_eq_ values were lower than current GC content in SC regions indicating that the GC content of SC regions was evolved toward AT-richness. On the contrary, the results show that GC_eq_ values of IRs (42.86–90.02% for protein-coding genes and 21.72–88.54% for non-coding loci) were higher than current GC content, suggesting that the evolution trends of GC content in IRs were toward GC-richness. Considering the consistent evolution trends between mutational biases and the evolution of GC content in SC and IRs, we proposed that mutational biases have directly impact on the evolution of GC content in *Dendrobium* plastomes.

### 2.6. gBGC Has an Effect on Maintaining Higher GC Content in IR Regions

Figure S4 illustrated the non-synonymous (dn) and synonymous (ds) substitution rates of protein-coding genes and mutation rates estimated from the syntenic non-coding loci in the SC and IR regions. The two-sided Mann–Whitney test indicated that the substitution rates were higher in SC regions than that in IR regions of *Dendrobium* plastomes (*p* < 0.01). The reduced substitution rates of IR regions most likely resulted from gene conversion. The intraplastomic recombination via IR regions frequently occurred in the plastid because of the identical sequences in the two IR regions (Figure S5A). Therefore, the GC-biased mutation and reduced substitution rates in IRs could be caused by gBGC. To determine whether the gBGC has an effect on the evolution of GC content, we compared the GC content of IR regions between the two groups: (1) the one-IR group, including five plastomes from Pinaceae and cupressophytes, which contain only one copy of IR region; and (2) the two-IR group, including 10 *Dendrobium* plastomes, which contain two copies of IR regions. Our comparison revealed a significantly higher GC content in the IR regions of the two-IR group than that of the one-IR group (Mann–Whitney 2-sides, *p* < 0.01), which suggested that the gBGC has influenced the GC content in IR regions (Figure S5B). Furthermore, the significant correlation between the GC_eq_ values and current GC content in protein-coding genes and non-coding regions (Spearman’s *r* = 0.851, 0.841, both *p* < 0.01) indicated that gBGC has a long-term effect on the GC content in *Dendrobium* plastomes.

## 3. Discussion

### 3.1. The High-Quality Complete Plastome Sequences That Could Represent for Dendrobium Species

Plastome sequences of seed plants, in general, have relatively small sizes, conserved gene contents, dense coding regions, and slower evolutionary rates as compared to nuclear and mitochondrial genomes [1]. These unique features have attracted intense attention from researchers, leading to numerous plastome sequences published, e.g., [20,22,25,26]. The first record of a *Dendrobium* plastome, *Dendrobium officinale*, was obtained by sequencing from the isolated chloroplast DNA [21]. However, it is extremely hard to extract the chloroplast from *Dendrobium* species due to their high percentage of polysaccharides, e.g., [27,28]. The plant whole genome sequencing data always contains high copy numbers of plastome sequence reads, which provide an opportunity for the complete plastome sequence assembly. Thus, a lot of methods were established to assemble the plastome sequence from the genome sequencing resources [29,30,31]. Among them, the reference-guided mapping method is a good approach for studies of related species with known reference sequences. Recently, more than 30 *Dendrobium* plastomes have been sequenced and assembled by using this approach [22]. However, tentative errors, such as SNP, InDel, and repeat sequences, may generate by using different assembly tools or mapping data to different references [31]. As shown in Figure S1, the sequences in the non-coding regions and *ndh* genes of *D. loddigesii* and *D. devonianum* were distinct from different assembly methods. Therefore, the high-quality plastome sequence that could be representing for *Dendrobium* species was urgently needed. In this study, two different methods were employed for the plastome assembly of *Dendrobium* species. According to the mVISTA analysis, the mis-assembled error sequences were detected, which indicated that at least two different methods should be used in the plastome assembly analysis to avoid assembling errors. After correcting and validating these errors by PCR amplification and Sanger sequencing, the complete plastomes of *D. loddigesii* and *D. devonianum* were obtained. Therefore, we proposed that the high-quality complete plastome sequences of *D. loddigesii* and *D. devonianum* were obtained in this study, which could represent for *Dendrobium* species for the future studies.

### 3.2. Both Mutational Biases and gBGC Affect GC Content in the Plastomes of Dendrobium Genus

The causes of GC content variation in plastome sequences were less clearly established than in nuclear genome sequences, and the role of gene conversion has only been investigated in non-coding regions more recently [17]. Previously, more than 30 plastomes of *Dendrobium* species have been published, which provides a huge resource for exploring the evolutionary mechanism of the variation of GC content in *Dendrobium* plastomes. Niu et al., (2017) proposed that evolutionary stasis should use at least 10 plastome sequences [22]. Here, we examined and discussed the three major hypotheses based on our newly sequenced data coupled with other eight *Dendrobium* plastomes.

#### 3.2.1. Natural Selection

Natural selection has great impact on the variations in base composition. For example, in plastomes, the codon-usage of the *psbA* gene was proposed to be associated with positive selection for maintaining the translation efficiency [32]. Moreover, several studies have suggested a correlation between GC content and the function and expression of genes e.g., [5,33,34], which enhanced the hypothesis that natural selection has a direct impact on GC content. The “natural selection hypothesis” was also supported by the study of Shi et al., (2007), who found that the selection is the primary cause of the GC content variation in rice genes [35]. In this study, positive selection analysis was performed for the 68 plastid protein-coding genes. The higher GC content in the IRs than that in SC regions leads us to expect that genes located in IRs have a higher proportion under positive selection. However, only one protein-coding gene (*ycf2*) was identified under positive selection in the IR regions. Moreover, the same mutational trends were observed in both positive selected and non-positive selected data sets. These results suggested that natural selection does not appear to contribute significantly to the varied GC content of plastid protein-coding genes. Therefore, we ruled out the effect of natural selection, which leaves the mutational bias and gBGC as potential determinants for the evolution of GC content in the *Dendrobium* plastomes.

#### 3.2.2. Mutational Bias

In this study, our analysis revealed a contrast mutational bias in *Dendrobium* plastomes that: (1) toward AT-richness in SC and (2) toward GC-richness in IR regions. The contrast mutational biases would lead to a lower GC content in SC regions. Indeed, the GC contents of LSC and SSC regions are lower than that of IRs. Moreover, the result estimated GC_eq_ values lower than current GC content in SC regions and higher than that of IRs, indicating that the GC content variation in SC regions is primarily determined by the mutational biases. Therefore, we proposed that mutational biases have direct impact on the variation of GC content in *Dendrobium* plastomes. Mutational biases toward AT-richness have been reported in gnetophytes [3]. Their plastomes were discovered to be compacting with enriched AT content, which was thought to have many advantages for resource consumption. For example, an A/T nucleotide contains seven atoms of nitrogen, one less than the G/C nucleotide. Moreover, AT-richness in base composition would benefit the rapid replication of plastomes [36]. Therefore, the sequence feature that mutated toward AT-richness would be favored in the plastome.

#### 3.2.3. gBGC

The variation in base composition is proven to be affected by gene conversion, e.g., [37,38]. The gene conversion of the plastome sequences is typically modeled as an independent process, in which changes at any one site are independent of changes at any other site [39]. However, gene conversion is not an entirely stochastic process, as it acts according to certain deterministic biases that affect the GC content evolution. Over the past 10 years, GC-biased gene conversion (gBGC) has been clearly established as the main process affecting GC content evolution in the nuclear genome [40,41,42,43]. However, whether the gBGC affects the GC content in plastome sequences is poorly understood. In this study, although no direct evidence is detected, we cannot ignore the impact of gBGC to the GC content evolution in *Dendrobium* genus. Based on the comparison results of 10 *Dendrobium* plastomes, we proposed that the gBGC has influenced the plastome wide GC content evolution due to three reasons. Firstly, the higher GC content in IR regions could be explained by gBGC. In this study, the reduced substitution rates and GC-biased mutation indicated that gBGC occurred in IRs. The gBGC would affect GC/AT heterozygous sites yields more frequently to GC than to AT alleles, which lead to increasing the GC content over evolutionary time [13]. Secondly, the recombination of IRs provides an opportunity for gBGC to occur. IRs are the recombination hotspots in plastome sequence, which favors the gBGC [44,45,46]. Moreover, a gBGC model during recombination has been put forth to interpret the GC-biased mutations observed in the IR regions [17]. Through that model, GC to AT mutations were unfavored, whereas AT to GC ones were fixed after gBGC. After loss of one IR region, the GC content of IR regions in Pinaceae and cupressophytes species was evolved toward AT-richness. Thirdly, the significant correlation between the GC_eq_ values and GC content values in protein-coding genes and non-coding regions (Spearman’s *r* = 0.851, 0.841, both *p* < 0.01) indicated that in *Dendrobium*, gBGC has shaped plastome-wide mutations and GC content. Therefore, we proposed that both mutational biases and gBGC affect GC content in the plastomes of the *Dendrobium* genus.

## 4. Materials and Methods

### 4.1. DNA Extraction and Plastome Sequencing

Two grams each of fresh leaves were harvested from individuals of *D. loddigesii* (voucher specimen: Niu14007) and *D. devonianum* (voucher specimen: Niu15008). They were cultivated in the greenhouse of Nanjing Normal University. The total DNA of each species was extracted using the Qiagen DNeasy Plant Mini Kit (Qiagen, Hilden, Germany), according to the manufacturer’s instructions. DNA samples that met the quality of concentrations >300 ng/mL, A260/A280 ratio = 1.8–2.0, and A260/A230 ratio >1.7, were used for next-generation sequencing. The sequencing depth was 3.84 GB of 150 bp paired-end reads for each species.

### 4.2. Plastome Assembly, Annotation and Comparison

To obtain high-quality complete plastome sequences of *D. loddigesii* and *D. devonianum*, two different assembly methods: (1) de novo assembly methods and (2) reference-guided mapping method, were employed in this study. For the de novo assembly, raw reads were first trimmed with error probability <0.05, then de novo assembled using SOAPdenovo version 1.12 with the default parameters [47]. The de Bruijn graph approach was applied to assembly with an optimal K-mer size of 79. Contigs with length <200 bp were discarded. The remaining contigs were compared with the plastome sequences of *D. moniliforme* (AB893950) using BLAST searches. Matched contigs with E-values of <10^−10^ were designated as plastomic contigs. The gaps between plastomic contigs were closed by PCR amplification with specific primers and Sanger sequencing. For reference-guided assembly, the trimmed reads were mapped to the reference plastome sequence of *D. moniliforme* (AB893950) using CLC Genomics Workbench 6.0.1 (CLC Bio, Aarhus, Denmark). The four junctions between LSC/SSC and IRs and the regions with uncertain nucleotide “N” were validated with PCR amplification. 

Protein-coding genes and ribosomal RNA genes were annotated using DOGMA [48]. The boundaries of each annotated gene were confirmed by their alignment with their orthologous genes from other *Dendrobium* plastomes. Genes of tRNA were predicted using tRNAscan-SE 1.21 [49]. The differences between plastome sequences of *D. loddigesii* and *D. devonianum* that assembled from different methods were compared by using the mVISTA software [50]. The plastome sequence of *D. moniliforme* (AB893950) was used as an outgroup.

### 4.3. Phylogenetic Analysis 

The plastome sequences of 10 *Dendrobium* species and *Phalaenopsis aphrodite* were aligned based on the MAFFT method [51]. The gaps within alignment were excluded. The phylogenetic tree was constructed using MrBayes 3.2 [52]. For the Bayesian inference (BI) analysis, two simultaneous runs were conducted, each consisting of four chains. The parameters were set as “lset nst = 6 rates = γ”. In total, chains were run for 2,000,000 generations, with topologies sampled every 100 generations. The first 25% of our sampled trees were discarded. The remaining trees were used to construct a majority-rule consensus tree and calculate posterior probabilities (PPs) for each node. 

### 4.4. Ancestral Non-Coding Sequences Reconstruction and Calculation of the Relative Rates of the Six Nucleotide Pair Mutations 

The non-coding loci, including intergenic and intronic loci, were manually retrieved from the 10 *Dendrobium* plastome sequences (Table S3). The loci were aligned by using MUSCLE [53] with the “Refining” option implemented in Mega 5.2 [54]. Gaps located at the 5′- and 3′-ends of alignments were excluded, and then concatenated using SequenceMatrix 1.8 [55]. The alignment of non-coding loci was divided into SC and IR data sets based on their locations. The ancestral non-coding sequences of 10 *Dendrobium* species were reconstructed using the maximum likelihood (ML) method with GTR + G model in Mega 5.2. The BI tree inferred from whole plastome sequences was designated as the “user tree”. DAMBE 5 [56] was used to count the nucleotide changes between ancestral and current sequences. After that, we calculated the relative rates of the six nucleotide pair mutations. Firstly, we normalized the counts of the mutations from A/T to G/C, C/G, or T/A, and followed Hershberg and Petrov’s method (2010) by multiplying them with current genome wide number of GC sites/AT sites [57]. For example, the normalized number of A to G mutations in *D. loddigesii* = the number of A to G changes between ancestral sequence and the current sequence of *D. loddigesii* × the current number of GC sites/AT sites. In this way, we could determine the expected number of mutations under equal GC and AT contents. Then, the relative rates of each nucleotide mutation were calculated with the formula = the number of mutations from G/C or the normalized number of mutations from A/T/(the total number of mutations from G/C + the normalized total number of mutations from A/T) × 100.

### 4.5. Estimation of GC_eq_

The GC_eq_ values were estimated according to the method of Hershberg and Petrov (2010) [57] and Wu et al., 2015 [17]. The GC_eq_ values were calculated as: rate of AT to GC/(rate of AT to GC + rate of GC to AT). The rate of AT to GC = the total number of AT to GC changes/the current number of AT sites, while the rate of GC to AT = the total number of GC to AT changes/the current number of GC sites. The SC and IR data sets including ancestral and current sequences were bootstrapped with 100 replications using PHYLIP 3.695 [58]. Then, the GC_eq_ values were recalculated 100 times and the resulting values were used to estimate the 95% confidence intervals for GC_eq_.

### 4.6. Natural Selection Analysis and Evolutionary Stasis of Protein-Coding Genes 

Except for the 11 *ndh* genes, positive selection analysis was performed for the other 68 protein-coding genes using the codeml program in PAML vs. 4.9 [59]. The natural selection at the codon level was detected using the three pairs of site models (M0 vs. M3, M1a vs. M2a, and M7 vs. M8) as implemented in codeml. The likelihood ratio tests (LRTs) were used to compare the site models. The relative rates of the six nucleotide pair mutations and GC_eq_ values of protein-coding genes were also counted.

### 4.7. Estimation of Substitution Rates

The synonymous (ds) and non-synonymous (dn) substitution rates were estimated with the codeml program of PAML [59]. The parameters were set to the following: seqtype = 1, runmodel = −2, and CodonFreq = 3. The protein-coding genes of *Phalaenopsis aphrodite* were used as the reference. The non-coding loci that flanked by the same genes/exons were identified as syntenic. The loci with sequence lengths less than 150 bp were discarded. The mutation rates between *P. aphrodite* and the 10 *Dendrobium* species were estimated with the baselml program of PAML using the REV model [59].

### 4.8. Statistical Analysis 

Statistical analyses were performed using SPSS Statistics 20.0. 

## 5. Conclusions

In conclusion, this study is the first to observe the impact of evolutionary forces, selection, mutational biases, and GC-biased gene conversion (gBGC) on the variation of GC content in the whole plastome sequences. The high-quality complete plastomes of *D. loddigesii* and *D. devonianum* were obtained and compared with eight other *Dendrobium* plastomes. The results indicate that the plastomes of *Dendrobium* species are highly conserved in plastomic organization. Because tentative errors generated by different assembly methods can lead to low quality of plastome sequence, we thus strongly suggest using two different assembly methods in the plastome assembly analysis. Furthermore, we examined three major hypotheses based on the plastome sequences of 10 *Dendrobium* species. Our results demonstrated that both mutational biases and gBGC affect GC content in the plastomes of the *Dendrobium* genus.

## Figures and Tables

**Table 1:** Genome feature of the two newly sequenced *Dendrobium* plastomes.

Species	Plastome Length (bp)	LSC Length (bp)	SSC Length (bp)	IR Length (bp)	GC Content (%)	GC Content of LSC (%)	GC Content of SSC (%)	GC Content of IR (%)	Accession
*Dendrobium loddigesii*	152,493	84,089	16,932	25,736	37.38	35.63	30.54	43.57	LC317044
*Dendrobium devonianum*	151,715	84,897	14,311	25,800	37.56	35.12	30.61	43.51	LC317045

Abbreviations: LSC, large single-copy; SSC, small single-copy; IR, inverted repeats.

**Table 2:** Summary of mutations in non-coding loci.

Species	SC		IR	
	Numbers of GC-AT Mutations	Numbers of Normalized AT-GC Mutations	Numbers of GC-AT Mutations	Numbers of Normalized AT-GC Mutations
*D. moniliforme*	229	66.65	6	7.74
*D. xichouense*	226	65.58	8	6.18
*D. officinale*	229	61.93	8	5.39
*D. falconeri*	248	76.54	6	4.63
*D. devonianum*	273	109.66	9	10.84
*D. primulinum*	221	60.78	8	7.71
*D. loddigesii*	210	62.25	13	8.62
*D. chrysotoxum*	188	61.10	1	7.74
*D. lohohense*	185	40.77	7	5.40
*D. salaccense*	452	104.40	14	6.92

**Table 3:** Summary of mutations in protein-coding loci.

Species	SC		IR	
	Numbers of GC-AT Mutations	Numbers of Normalized AT-GC Mutations	Numbers of GC-AT Mutations	Numbers of Normalized AT-GC Mutations
*D. moniliforme*	79	44.73	1	7.73
*D. xichouense*	115	45.95	8	5.78
*D. officinale*	100	51.09	8	10.29
*D. falconeri*	94	49.20	2	9.66
*D. devonianum*	89	53.71	5	5.79
*D. primulinum*	94	49.86	7	6.43
*D. loddigesii*	86	46.03	5	5.79
*D. chrysotoxum*	152	36.24	13	1.28
*D. lohohense*	59	48.04	3	5.79
*D. salaccense*	57	42.26	3	5.79

## Supplementary Materials

Supplementary materials can be found at www.mdpi.com1422-0067/18/11/2307/s1.
