# Supplementary material for: Mutational Biases and GC-Biased Gene Conversion Affect GC Content in the Plastomes of Dendrobium Genus
Source: Int J Mol Sci. 2017 Nov 2;18(11):2307. doi: 10.3390/ijms18112307 (PMC5713276; doi:10.3390/ijms18112307)
Supplement: Supplementary file 1 [file ijms-18-02307-s001.doc]

**Supplementary materials:**


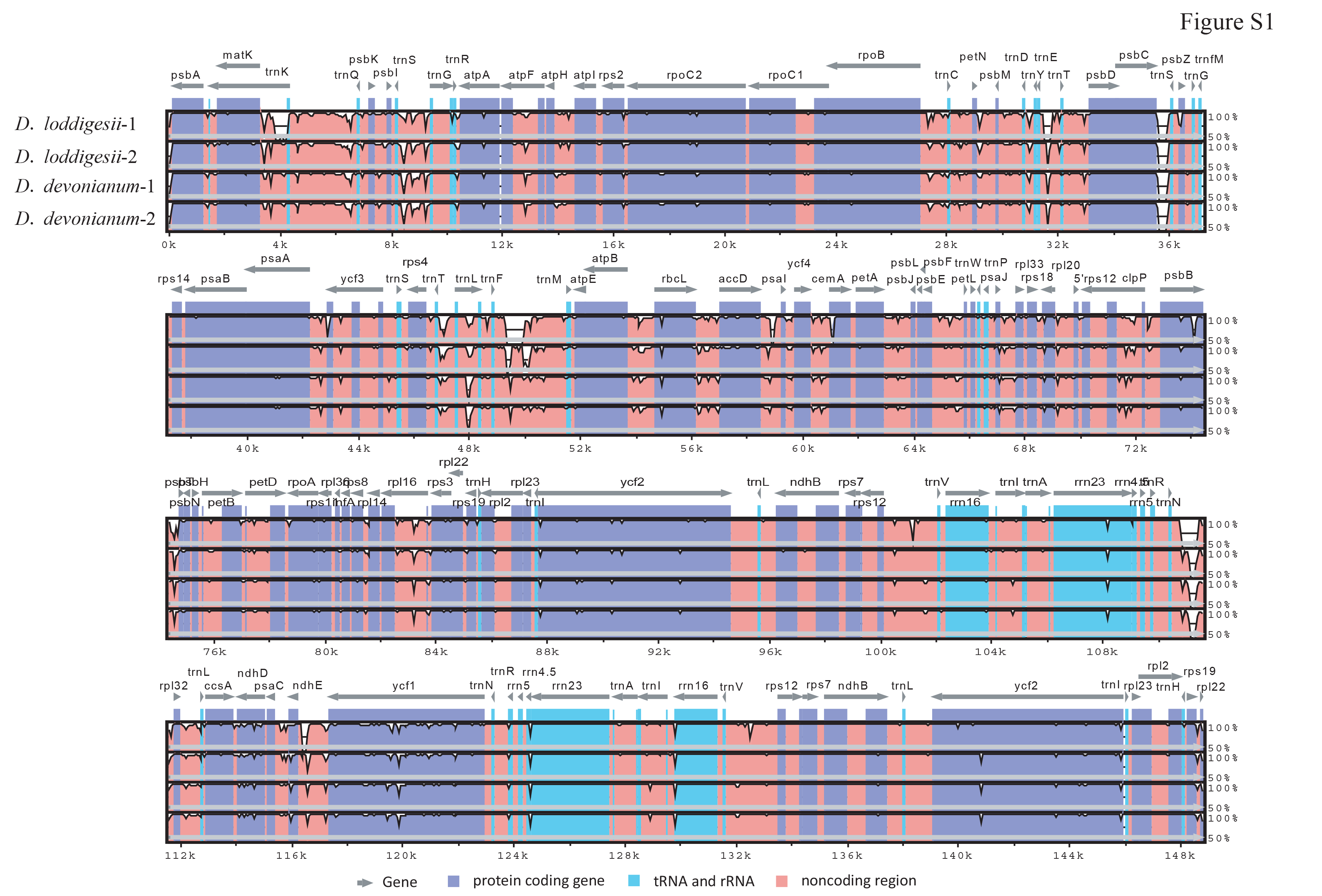


Figure S1: Sequence identity plots among the plastomes assembled from different methods with the plastome sequence *D. moniliforme* (AB893950) of as the reference. The plastomes of *D. loddigesii*-1 and *D. devonianum*-1 were assembled using *de novo* assemble methods. The plastomes of *D. loddigesii*-2 and *D. devonianum*-2 were assembled using reference-guided mapping method.


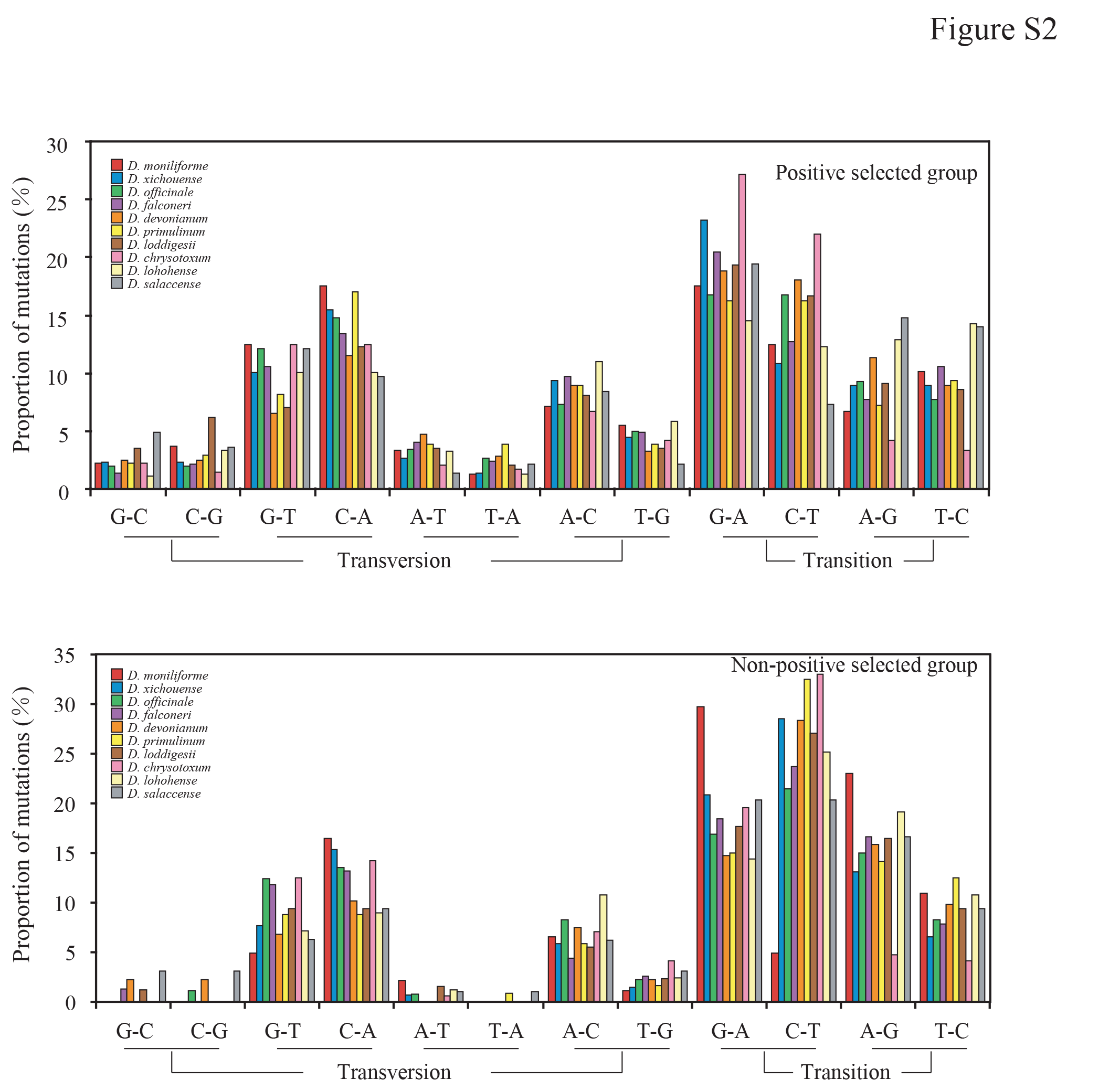


Figure S2: Proportion of the six nucleotide-pair mutations estimated from the protein-coding genes in positive selected and non-positive selected groups.


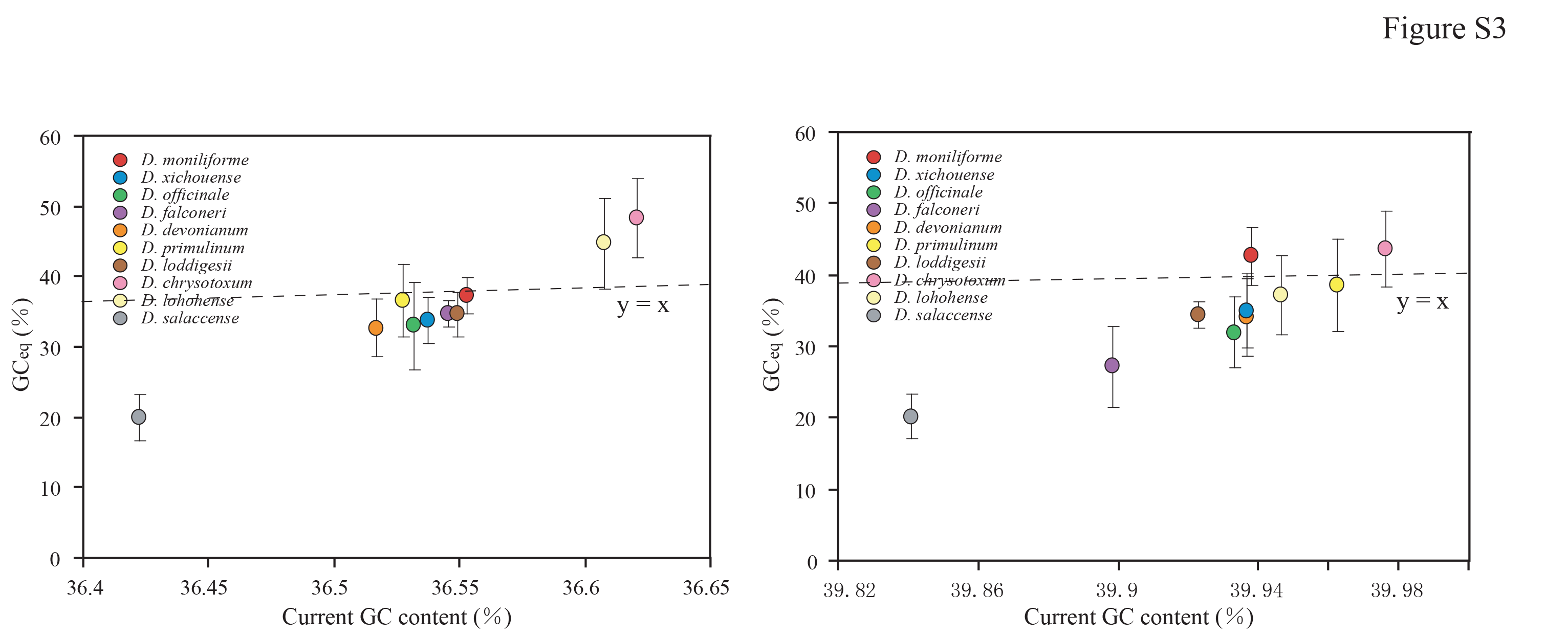


Figure S3: GC_eq_ values between the protein-coding genes in positive selected and non-positive selected groups.


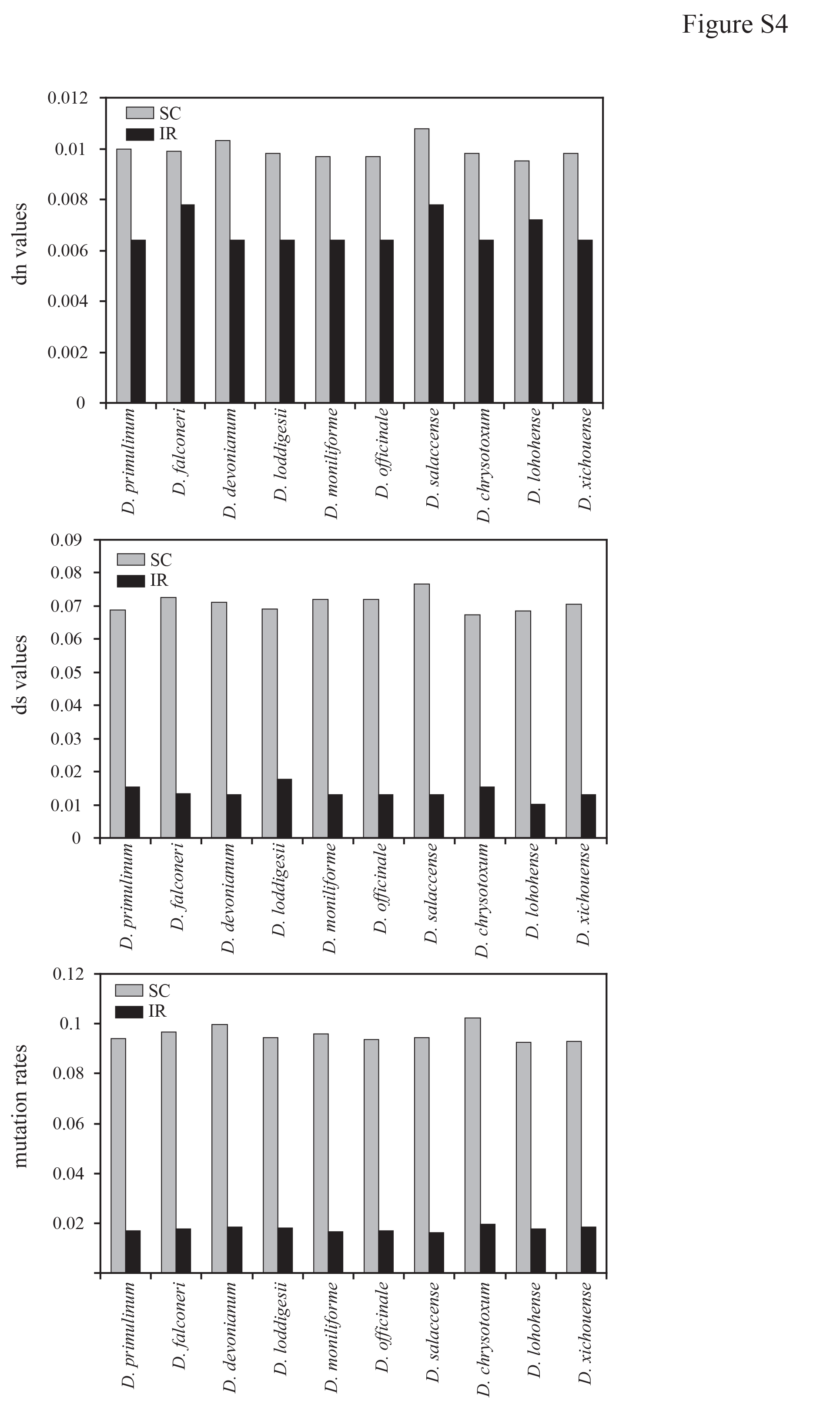


Figure S4: The non-synonymous (dn) and synonymous (ds) substitution rates of protein-coding genes and mutation rates estimated from the syntenic non-coding loci in the SC and IR regions.


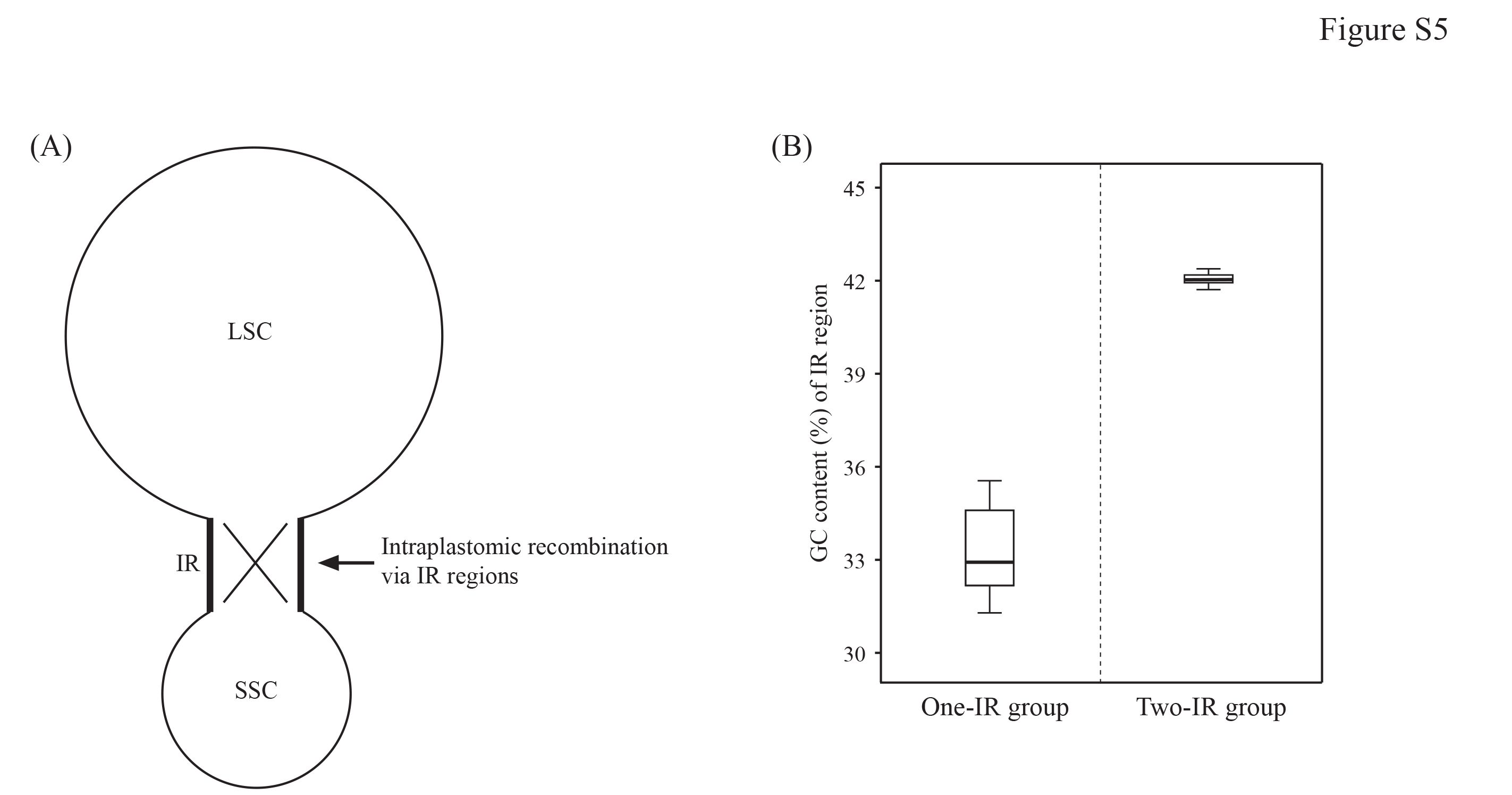


Figure S5: (A) The model of intraplastomic recombination via IR regions. (B) The comparison the GC content of IR regions between the two groups: (1) one-IR group, including five plastomes from Pinaceae and cupressophytes, which contain only one copy of IR region; (2) two-IR group, including ten *Dendrobium* plastomes, which cotain two copy of IR regions.

| Table S1 Estimate of log likelihood values under three pairs of site models. | | | | | |
| --- | --- | --- | --- | --- | --- |
| Gene |  | LRT |  | 2△lnL | *P* |
| *accD* | M0 | vs | M3 | 26.36367 | 0.000026726 |
|  | M1a | vs | M2a | 6.144006 | 0.046328266 |
|  | M7 | vs | M8 | 6.442872 | 0.039897724 |
| *ccsA* | M0 | vs | M3 | 13.92282 | 0.007545487 |
|  | M1a | vs | M2a | 6.796042 | 0.033439381 |
|  | M7 | vs | M8 | 8.202774 | 0.016549705 |
| *matK* | M0 | vs | M3 | 53.68709 | 0 |
|  | M1a | vs | M2a | 24.76618 | 0.000004189 |
|  | M7 | vs | M8 | 24.86317 | 0.000003991 |
| *psaB* | M0 | vs | M3 | 18.19516 | 0.001130286 |
|  | M1a | vs | M2a | 3.756018 | 0.152894215 |
|  | M7 | vs | M8 | 11.70186 | 0.002877228 |
| *rbcL* | M0 | vs | M3 | 97.34326 | 0 |
|  | M1a | vs | M2a | 47.39823 | 0 |
|  | M7 | vs | M8 | 54.09015 | 0 |
| *rpl20* | M0 | vs | M3 | 33.03816 | 0.000001173 |
|  | M1a | vs | M2a | 13.85973 | 0.000978134 |
|  | M7 | vs | M8 | 18.10779 | 0.000116934 |
| *rpoC1* | M0 | vs | M3 | 23.50779 | 0.000100228 |
|  | M1a | vs | M2a | 3.367564 | 0.185670441 |
|  | M7 | vs | M8 | 9.509404 | 0.00861111 |
| *rpoC2* | M0 | vs | M3 | 28.27698 | 0.00001096 |
|  | M1a | vs | M2a | 10.25085 | 0.005943685 |
|  | M7 | vs | M8 | 11.38209 | 0.00337606 |
| *rps3* | M0 | vs | M3 | 18.6978 | 0.000900985 |
|  | M1a | vs | M2a | 4.250858 | 0.119381743 |
|  | M7 | vs | M8 | 9.670002 | 0.00794668 |
| *rps16* | M0 | vs | M3 | 32.32307 | 0.000001643 |
|  | M1a | vs | M2a | 10.17279 | 0.006180253 |
|  | M7 | vs | M8 | 10.20044 | 0.006095405 |
| *ycf1* | M0 | vs | M3 | 56.00589 | 0 |
|  | M1a | vs | M2a | 6.521448 | 0.038360615 |
|  | M7 | vs | M8 | 16.64663 | 0.000242789 |
| *ycf2* | M0 | vs | M3 | 26.17892 | 0.000029121 |
|  | M1a | vs | M2a | 20.23973 | 0.000040271 |
|  | M7 | vs | M8 | 20.17841 | 0.000041525 |
|  | | | | | |

| Table S2 Summary of mutations in positive selected and non-positive selected group. | | | | |
| --- | --- | --- | --- | --- |
| Species | Positive selected group | | Non-positive selected group | |
|  | Numbers of GC-AT mutations | Numbers of normalized AT-GC mutations | Numbers of GC-AT mutations | Numbers of normalized AT-GC mutations |
| *D. moniliforme* | 82 | 40.29 | 34 | 25.27 |
| *D. xichouense* | 77 | 40.89 | 66 | 24.56 |
| *D. officinale* | 90 | 43.72 | 57 | 29.90 |
| *D. falconeri* | 81 | 46.61 | 51 | 23.93 |
| *D. devonianum* | 67 | 39.75 | 53 | 31.26 |
| *D. primulinum* | 78 | 39.73 | 52 | 27.26 |
| *D. loddigesii* | 63 | 33.41 | 54 | 28.59 |
| *D. chrysotoxum* | 101 | 25.21 | 89 | 22.52 |
| *D. lohohense* | 42 | 39.29 | 31 | 23.98 |
| *D. salaccense* | 40 | 32.34 | 36 | 22.63 |
|  | | | | |

| Table S3 Taxa and their GenBank accession numbers used in this study. | | |
| --- | --- | --- |
| Genus | Taxon | Accession |
| *Dendrobium* | *D. loddigesii*^*^ | LC317044 |
|  | *D. devonianum*^*^ | LC317045 |
|  | *D. moniliforme* | AB893950 |
|  | *D. officinale* | NC_024019 |
|  | *D. xichouense* | LC193520 |
|  | *D. lohohense* | LC193516 |
|  | *D. chrysotoxum* | LC193517 |
|  | *D. primulinum* | LC192810 |
|  | *D. salaccense* | LC193510 |
|  | *D. falconeri* | LC192957 |
| *Phalaenopsis* | *P. aphrodite* | NC_007499 |
| ^*^: plasotmes sequenced in this study | | |
